# Theobrominium perchlorate dibenzo-18-crown-6 3.25-hydrate

**DOI:** 10.1107/S1600536813014463

**Published:** 2013-06-08

**Authors:** Vladislav Kulikov, Gerd Meyer

**Affiliations:** a Institut für Anorganische Chemie, Universität zu Köln, Greinstrasse 6, D-50939 Köln, Germany

## Abstract

The co-crystal, C_7_H_9_N_4_O_2_^+^·ClO_4_^−^·C_20_H_24_O_6_·3.25H_2_O, consists of theobrominium (3,7-di­methyl-2,6-dioxo-1*H*-purin-9-ium) cations, perchlorate anions and dibenzo-18-crown-6 and water mol­ecules. The crown ether is in a bent conformation, in which the planes of the aromatic rings subtend an angle of 63.7 (1)°. Inter­molecular O—H⋯O hydrogen bonding between the water mol­ecules and the O atoms of the cyclic ether delimit an empty space reminiscent of a hollow cage. The water mol­ecules are additionally linked to the cations by N—H⋯O hydrogen bonding. One of the positions of the water molecules is occupied only fractionally (25%) and is located outside this framework.

## Related literature

For applications of crown ethers, see: Lehn (1995 ▶). For host–guest chemistry of dibenzo-18-crown-6 with nitrogen bases, see: Lämsä *et al.* (1998 ▶). For the crystal structure of dibenzo-18-crown-6, see: Lima *et al.* (2008 ▶).
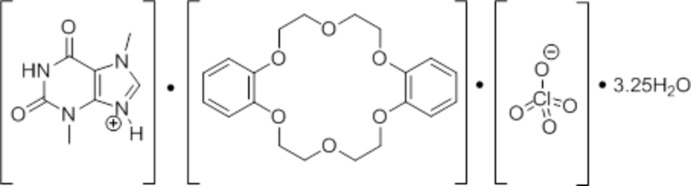


## Experimental

### 

#### Crystal data

C_7_H_9_N_4_O_2_^+^·ClO_4_^−^·C_20_H_24_O_6_·3.23H_2_O
*M* *_r_* = 699.58
Orthorhombic, 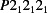

*a* = 11.9292 (3) Å
*b* = 15.2505 (5) Å
*c* = 18.1222 (5) Å
*V* = 3296.90 (16) Å^3^
*Z* = 4
Mo *K*α radiation
μ = 0.19 mm^−1^
*T* = 293 K
0.5 × 0.5 × 0.3 mm

#### Data collection

Stoe & Cie IPDS II diffractometer
Absorption correction: numerical [*X-RED* (Stoe & Cie, 2001 ▶) and *X-SHAPE* (Stoe & Cie, 1999 ▶)] *T*_min_ = 0.991, *T*_max_ = 0.997
51074 measured reflections
7018 independent reflections
5299 reflections with *I* > 4σ(*I*)
*R*_int_ = 0.084

#### Refinement

*R*[*F* ^2^ > 2σ(*F* ^2^)] = 0.049
*wR*(*F* ^2^) = 0.156
*S* = 1.04
7017 reflections
434 parameters
H-atom parameters constrained
Δρ_max_ = 0.32 e Å^−3^
Δρ_min_ = −0.33 e Å^−3^
Absolute structure: Flack (1983 ▶), 3092 Friedel pairs
Flack parameter: 0.02 (10)

### 

Data collection: *X-AREA* (Stoe & Cie, 2002 ▶); cell refinement: *X-AREA*; data reduction: *X-AREA*; program(s) used to solve structure: *SIR92* (Altomare *et al.*, 1993 ▶); program(s) used to refine structure: *SHELXL97* (Sheldrick, 2008 ▶); molecular graphics: *DIAMOND* (Crystal Impact, 2012 ▶); software used to prepare material for publication: *SHELXL97*.

## Supplementary Material

Crystal structure: contains datablock(s) global, I. DOI: 10.1107/S1600536813014463/nc2312sup1.cif

Structure factors: contains datablock(s) I. DOI: 10.1107/S1600536813014463/nc2312Isup2.hkl

Additional supplementary materials:  crystallographic information; 3D view; checkCIF report

## Figures and Tables

**Table 1:** Hydrogen-bond geometry (Å, °)

*D*—H⋯*A*	*D*—H	H⋯*A*	*D*⋯*A*	*D*—H⋯*A*
N9—H9⋯O3*W*	0.86	1.74	2.594 (4)	172
O1*W*—H1*O*1⋯O12	0.82	2.17	2.941 (3)	158
O2*W*—H1*O*2⋯O16	0.82	2.41	3.156 (3)	152
O2*W*—H1*O*2⋯O11	0.82	2.48	3.182 (3)	145
O2*W*—H2*O*2⋯O14	0.82	2.31	3.124 (3)	175
O3*W*—H1*O*3⋯O1*W*	0.82	1.99	2.802 (3)	172
O3*W*—H2*O*3⋯O15	0.82	1.98	2.769 (3)	161
N1—H1⋯O1*W* ^i^	0.86	2.01	2.871 (3)	174
O1*W*—H2*O*1⋯O2*W* ^ii^	0.82	1.93	2.747 (3)	175

Symmetry codes: (i) 
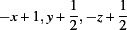
; (ii) 
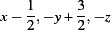
.

**Fig. 1.**
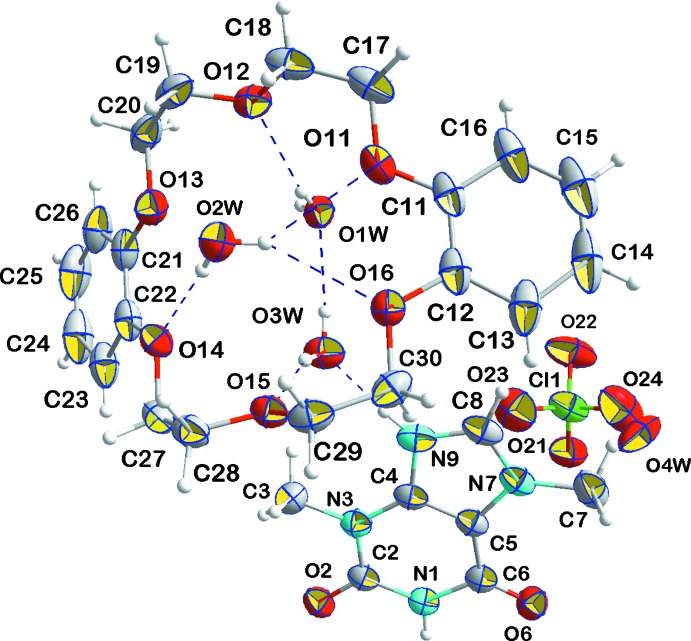
Crystal structure of the title compound with labeling and displacement ellipsoids drawn at the 30% probability level. H atoms are shown as small spheres of arbitrary radii and intermolecular hydrogen bonding is shown as dashed lines.

**Fig. 2.**
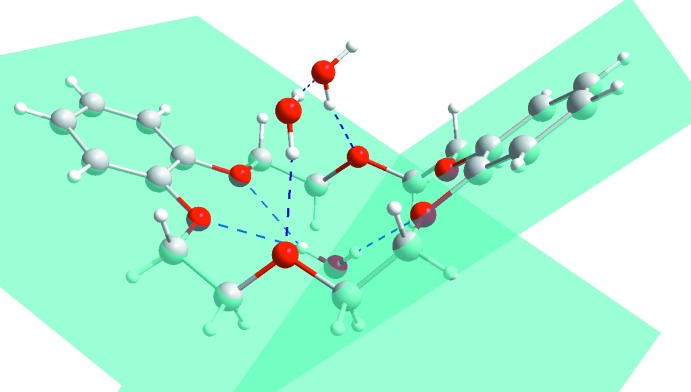
View of the dibenzo-18-crown-6 molecule and the co-crystallizing water molecules with intermolecular O—H···O hydrogen bonding shwon as dashed lines.

## supplementary crystallographic information

### Comment

Benzene substituted crown ethers have been instrumental for the development of supramolecular chemistry (Lehn, 1995). Among other areas, biological implications attracted scientific interest, due to the vital importance of host–guest interactions for biological processes (Lämsä *et al.*, 1998 and references therein). Theobromine is one of the biomolecules that are likely to interact with bioreceptors which show some similarities with crown ethers.

In the crystal structure of the title compound the dibenzo-18-crown-6 molecule is in the usual bent conformation (Fig. 1) The angle between the planes of the aromatic rings is 63.7 (1)°, which is slightly lower than the one reported for the crystal structure of the neat molecule (Lima *et al.*, 2008). The oxygen atoms of the ether build hydrogen bonds with two water molecules above (O1W and O3W) and one below (O2W) the central part of the ring (Fig. 2). The resulting geometric arrangement is reminiscent of a hollow cage with O-atoms on the vertices and H-bonds defining the sides. The "cages" are interlinked with one another *via* H-bonds between water molecules. The theobrominium ions are connected to the H-bonding framework *via* intermolecular N—H···O hydrogen bonding between N9 and O3W (Table 1).

The pyrimidine ring of the theobromine molecule appears to be superimposed over one of the aromatic rings of dibenzo-18-crown-6. The angle enclosed by the planes of the purine and benzene ring is 9.18 (8)°. Due to this relatively large value of the interplanar angle, π-π stacking interactions between both aromatic moieties appear to be unlikely.

### Experimental

Theobromine (18 mg, 0.1 mmol) was dissolved in aqueous HClO_4_ solution (5.6 ml, 6.4%) and added to the suspension of AgClO_4_ (20 mg, 0.1 mmol) and dibenzo-18-crown-6 (36 mg, 0.1 mmol) in a mixture of toluene (6.3 ml) and dichloromethane (0.3 ml). The biphasic suspension was stirred vigorously for 1.5 hrs. and filtered. After 6 weeks of slow solvent evaporation at room temperature and several cycles of filtration the mother liquor was cooled for 10 days at 4°C. One colourless pentagonal prismatic crystal could be isolated among thin colourless intergrown needles.

### Refinement

All C—H and N—H H atoms were positioned with idealized geometry and were refined isotropic with *U*_iso_(H) = 1.2 *U*_eq_(C, N) (1.5 for methyl H atoms) using a riding model with C - H = 0.970 Å for methylene, 0.930 Å for aromatic, 0.97 Å for methyl and 0.86 Å for N—H H atoms. The O—H H atoms of the water molecules at O1W, O2W and O3W were located in difference map, their bond lengths were set to 0.82 Å and afterwards they were refined isotropic with *U*_iso_(H) = 1.5 *U*_eq_(O) using a riding model. The position of the water molecule O4W is occupied to only 25% and its H atoms were not located.

### Crystal data

**Table:**

C_7_H_9_N_4_O_2_^+^·ClO_4_^−^·C_20_H_24_O_6_·3.23H_2_O	*F*(000) = 1472
*M**_r_* = 699.58	*D*_x_ = 1.412 Mg m^−^^3^
Orthorhombic, *P*2_1_2_1_2_1_	Mo *K*α radiation, λ = 0.71073 Å
Hall symbol: P 2ac 2ab	Cell parameters from 51177 reflections
*a* = 11.9292 (3) Å	θ = 0.8–27.4°
*b* = 15.2505 (5) Å	µ = 0.19 mm^−^^1^
*c* = 18.1222 (5) Å	*T* = 293 K
*V* = 3296.90 (16) Å^3^	Pentagonal prism, colourless
*Z* = 4	0.5 × 0.5 × 0.3 mm

### Data collection

**Table:**

Stoe & Cie IPDS II diffractometer	7018 independent reflections
Radiation source: fine-focus sealed tube	5299 reflections with *I* > 4σ(*I*)
Graphite monochromator	*R*_int_ = 0.084
ω and φ scans	θ_max_ = 26.8°, θ_min_ = 2.4°
Absorption correction: numerical [*X-RED* (Stoe & Cie, 2001) and *X-SHAPE* (Stoe & Cie, 1999)]	*h* = −14→15
*T*_min_ = 0.991, *T*_max_ = 0.997	*k* = −19→19
51074 measured reflections	*l* = −22→22

### Refinement

**Table:**

Refinement on *F*^2^	Hydrogen site location: inferred from neighbouring sites
Least-squares matrix: full	H-atom parameters constrained
*R*[*F*^2^ > 2σ(*F*^2^)] = 0.049	*w* = 1/[σ^2^(*F*_o_^2^) + (0.0909*P*)^2^ + 0.3494*P*] where *P* = (*F*_o_^2^ + 2*F*_c_^2^)/3
*wR*(*F*^2^) = 0.156	(Δ/σ)_max_ < 0.001
*S* = 1.04	Δρ_max_ = 0.32 e Å^−^^3^
7017 reflections	Δρ_min_ = −0.33 e Å^−^^3^
434 parameters	Extinction correction: *SHELXL97* (Sheldrick, 2008), Fc^*^=kFc[1+0.001xFc^2^λ^3^/sin(2θ)]^-1/4^
0 restraints	Extinction coefficient: 0.0142 (14)
Primary atom site location: structure-invariant direct methods	Absolute structure: Flack (1983), 3092 Friedel pairs
Secondary atom site location: difference Fourier map	Flack parameter: 0.02 (10)

### Special details

**Table:**

Experimental. Absorption correction: The absorption correction (X-RED; Stoe & Cie, 2001) was performed after optimizing the crystal shape using X-SHAPE (Stoe & Cie, 1999). A suitable single-crystal was carefully selected under a polarizing microscope and mounted in a glass capillary. The scattering intensities were collected on an imaging plate diffractometer (*IPDS* II, Stoe & Cie) equipped with a fine focus sealed tube X-ray source (Mo Kα, λ = 0.71073 Å) operating at 50 kV and 40 mA. Intensity data for [C_7_H_9_N_4_O_2_]^+^ [ClO_4_]^-.^ C_20_H_24_O_6_^.^ (H_2_O)_3.25_ were collected at 170 K by ω-scans in 360 frames (0 < ω < 180°; φ = O°, 0 < ω < 180°; φ = 90°, exposure time of 5 min) in the 2 Θ range 4.88 to 54.41°.
Geometry. All e.s.d.'s (except the e.s.d. in the dihedral angle between two l.s. planes) are estimated using the full covariance matrix. The cell e.s.d.'s are taken into account individually in the estimation of e.s.d.'s in distances, angles and torsion angles; correlations between e.s.d.'s in cell parameters are only used when they are defined by crystal symmetry. An approximate (isotropic) treatment of cell e.s.d.'s is used for estimating e.s.d.'s involving l.s. planes.
Refinement. Refinement of *F*^2^ against ALL reflections. The weighted *R*-factor *wR* and goodness of fit *S* are based on *F*^2^, conventional *R*-factors *R* are based on *F*, with *F* set to zero for negative *F*^2^. The threshold expression of *F*^2^ > σ(*F*^2^) is used only for calculating *R*-factors(gt) *etc*. and is not relevant to the choice of reflections for refinement. *R*-factors based on *F*^2^ are statistically about twice as large as those based on *F*, and *R*- factors based on ALL data will be even larger.

### Fractional atomic coordinates and isotropic or equivalent isotropic displacement parameters (Å^2^)

**Table:**

	*x*	*y*	*z*	*U*_iso_*/*U*_eq_	Occ. (<1)
O2	0.4670 (2)	1.15212 (15)	0.28756 (13)	0.0795 (6)	
O6	0.4096 (2)	0.96464 (16)	0.47863 (13)	0.0807 (6)	
N1	0.44307 (18)	1.05635 (15)	0.38208 (14)	0.0625 (5)	
H1	0.4110	1.0977	0.4065	0.075*	
N3	0.5349 (2)	1.01361 (16)	0.27257 (14)	0.0660 (6)	
N7	0.5362 (2)	0.82655 (16)	0.38593 (16)	0.0726 (6)	
N9	0.5930 (2)	0.85970 (17)	0.27451 (16)	0.0743 (7)	
H9	0.6221	0.8547	0.2313	0.089*	
C2	0.4802 (2)	1.07851 (18)	0.31248 (17)	0.0635 (6)	
C3	0.5843 (4)	1.0351 (3)	0.20151 (19)	0.0875 (9)	
H3A	0.5502	1.0875	0.1825	0.131*	
H3B	0.5720	0.9877	0.1677	0.131*	
H3C	0.6634	1.0445	0.2074	0.131*	
C4	0.5461 (2)	0.93354 (18)	0.30451 (17)	0.0627 (6)	
C5	0.5097 (2)	0.91390 (18)	0.37377 (16)	0.0625 (6)	
C6	0.4505 (2)	0.97669 (19)	0.41781 (17)	0.0633 (6)	
C7	0.5171 (4)	0.7767 (2)	0.4533 (2)	0.0985 (12)	
H7A	0.4483	0.7955	0.4758	0.148*	
H7B	0.5781	0.7862	0.4869	0.148*	
H7C	0.5122	0.7154	0.4416	0.148*	
C8	0.5846 (3)	0.7971 (2)	0.3256 (2)	0.0798 (9)	
H8	0.6098	0.7399	0.3194	0.096*	
O11	0.88091 (18)	0.59317 (13)	0.11303 (14)	0.0735 (5)	
O12	0.85891 (16)	0.62645 (15)	−0.03842 (12)	0.0727 (5)	
O13	0.83609 (19)	0.80905 (15)	−0.06969 (11)	0.0743 (5)	
O14	0.85961 (18)	0.92968 (13)	0.02745 (12)	0.0702 (5)	
O15	0.90850 (16)	0.89503 (13)	0.17826 (12)	0.0663 (5)	
O16	0.9051 (2)	0.71309 (14)	0.21114 (11)	0.0718 (5)	
C11	0.8407 (2)	0.5724 (2)	0.1810 (2)	0.0746 (8)	
C12	0.8526 (3)	0.6374 (2)	0.2340 (2)	0.0768 (9)	
C13	0.8139 (3)	0.6234 (3)	0.3055 (2)	0.1026 (14)	
H13	0.8210	0.6659	0.3421	0.123*	
C14	0.7622 (4)	0.5399 (4)	0.3200 (3)	0.121 (2)	
H14	0.7349	0.5283	0.3671	0.146*	
C15	0.7523 (4)	0.4778 (4)	0.2674 (4)	0.127 (2)	
H15	0.7194	0.4241	0.2782	0.153*	
C16	0.7903 (3)	0.4945 (2)	0.1998 (3)	0.0981 (13)	
H16	0.7824	0.4515	0.1637	0.118*	
C17	0.8778 (3)	0.5258 (2)	0.0583 (2)	0.0854 (10)	
H17A	0.8008	0.5091	0.0482	0.102*	
H17B	0.9181	0.4745	0.0755	0.102*	
C18	0.9306 (3)	0.5608 (2)	−0.0088 (2)	0.0851 (10)	
H18A	1.0032	0.5859	0.0030	0.102*	
H18B	0.9414	0.5143	−0.0445	0.102*	
C19	0.9059 (3)	0.6708 (3)	−0.10055 (17)	0.0792 (9)	
H19A	0.9171	0.6296	−0.1406	0.095*	
H19B	0.9781	0.6954	−0.0874	0.095*	
C20	0.8298 (3)	0.7414 (3)	−0.12458 (16)	0.0803 (9)	
H20A	0.8528	0.7639	−0.1723	0.096*	
H20B	0.7537	0.7196	−0.1286	0.096*	
C21	0.7786 (2)	0.8836 (2)	−0.08359 (18)	0.0740 (8)	
C22	0.7899 (2)	0.9493 (2)	−0.03031 (19)	0.0736 (8)	
C23	0.7343 (3)	1.0296 (3)	−0.0379 (2)	0.0947 (12)	
H23	0.7404	1.0735	−0.0026	0.114*	
C24	0.6683 (4)	1.0404 (4)	−0.1022 (4)	0.125 (2)	
H24	0.6303	1.0930	−0.1091	0.150*	
C25	0.6586 (4)	0.9765 (5)	−0.1541 (3)	0.130 (2)	
H25	0.6151	0.9859	−0.1959	0.155*	
C26	0.7125 (3)	0.8991 (3)	−0.1449 (2)	0.0967 (12)	
H26	0.7049	0.8555	−0.1804	0.116*	
C27	0.8772 (3)	0.99687 (19)	0.0821 (2)	0.0762 (8)	
H27A	0.8072	1.0107	0.1067	0.091*	
H27B	0.9055	1.0498	0.0590	0.091*	
C28	0.9597 (3)	0.9629 (2)	0.13603 (19)	0.0747 (8)	
H28A	1.0247	0.9400	0.1103	0.090*	
H28B	0.9841	1.0098	0.1684	0.090*	
C29	0.9845 (3)	0.8518 (2)	0.22719 (18)	0.0738 (8)	
H29A	1.0125	0.8930	0.2635	0.089*	
H29B	1.0479	0.8290	0.1996	0.089*	
C30	0.9255 (3)	0.7794 (2)	0.26431 (17)	0.0782 (8)	
H30A	0.9710	0.7564	0.3042	0.094*	
H30B	0.8552	0.8002	0.2848	0.094*	
Cl1	0.22252 (9)	0.73830 (6)	0.35970 (7)	0.1009 (3)	
O21	0.1452 (4)	0.7997 (2)	0.3897 (2)	0.1286 (11)	
O22	0.1621 (4)	0.6775 (2)	0.3156 (3)	0.1529 (16)	
O23	0.3015 (3)	0.7825 (3)	0.3166 (3)	0.1486 (14)	
O24	0.2795 (4)	0.6895 (3)	0.4140 (3)	0.171 (2)	
O1W	0.67017 (17)	0.69899 (13)	0.04627 (11)	0.0677 (5)	
H1O1	0.7331	0.6867	0.0312	0.102*	
H2O1	0.6246	0.7115	0.0141	0.102*	
O2W	1.00739 (19)	0.76413 (15)	0.05640 (12)	0.0791 (6)	
H1O2	0.9760	0.7346	0.0881	0.119*	
H2O2	0.9660	0.8065	0.0507	0.119*	
O3W	0.69410 (18)	0.83435 (16)	0.14960 (13)	0.0825 (6)	
H1O3	0.6811	0.7941	0.1208	0.128*	
H2O3	0.7613	0.8457	0.1496	0.124*	
O4W	0.7438 (9)	0.6622 (7)	0.4630 (4)	0.093 (3)	0.25

### Atomic displacement parameters (Å^2^)

**Table:**

	*U*^11^	*U*^22^	*U*^33^	*U*^12^	*U*^13^	*U*^23^
O2	0.0822 (14)	0.0664 (12)	0.0901 (15)	0.0001 (10)	−0.0082 (11)	0.0057 (11)
O6	0.0735 (12)	0.0897 (15)	0.0787 (14)	−0.0036 (11)	0.0036 (11)	0.0004 (11)
N1	0.0560 (11)	0.0606 (12)	0.0708 (13)	0.0022 (10)	−0.0049 (10)	−0.0053 (10)
N3	0.0592 (12)	0.0669 (14)	0.0718 (14)	−0.0034 (10)	−0.0010 (10)	−0.0057 (11)
N7	0.0647 (13)	0.0625 (13)	0.0906 (17)	−0.0016 (11)	−0.0157 (13)	−0.0026 (13)
N9	0.0607 (13)	0.0712 (15)	0.0910 (17)	0.0033 (12)	−0.0082 (12)	−0.0223 (14)
C2	0.0523 (13)	0.0614 (15)	0.0767 (17)	−0.0024 (11)	−0.0096 (12)	−0.0057 (13)
C3	0.104 (2)	0.085 (2)	0.0738 (19)	−0.007 (2)	0.0098 (18)	−0.0055 (16)
C4	0.0486 (12)	0.0618 (15)	0.0777 (17)	−0.0023 (11)	−0.0086 (12)	−0.0121 (13)
C5	0.0542 (13)	0.0585 (14)	0.0749 (17)	−0.0012 (11)	−0.0100 (12)	−0.0035 (12)
C6	0.0496 (12)	0.0694 (16)	0.0710 (17)	−0.0045 (11)	−0.0085 (12)	−0.0038 (13)
C7	0.109 (3)	0.075 (2)	0.111 (3)	−0.007 (2)	−0.023 (2)	0.021 (2)
C8	0.0647 (16)	0.0633 (17)	0.111 (3)	0.0032 (14)	−0.0195 (18)	−0.0124 (18)
O11	0.0724 (12)	0.0569 (10)	0.0911 (14)	−0.0054 (9)	−0.0053 (11)	0.0116 (10)
O12	0.0560 (10)	0.0762 (12)	0.0859 (13)	0.0014 (9)	0.0037 (10)	−0.0127 (10)
O13	0.0752 (12)	0.0826 (13)	0.0653 (11)	−0.0069 (11)	−0.0087 (10)	0.0054 (10)
O14	0.0688 (11)	0.0595 (10)	0.0823 (12)	0.0028 (9)	−0.0039 (10)	0.0050 (9)
O15	0.0538 (9)	0.0670 (11)	0.0782 (11)	−0.0086 (8)	−0.0064 (9)	−0.0077 (9)
O16	0.0812 (12)	0.0683 (11)	0.0659 (11)	−0.0003 (10)	0.0046 (10)	0.0055 (9)
C11	0.0516 (13)	0.0677 (17)	0.105 (2)	0.0049 (12)	−0.0009 (15)	0.0291 (17)
C12	0.0557 (14)	0.082 (2)	0.093 (2)	0.0131 (14)	0.0066 (14)	0.0339 (18)
C13	0.077 (2)	0.125 (3)	0.106 (3)	0.032 (2)	0.022 (2)	0.057 (3)
C14	0.071 (2)	0.148 (4)	0.145 (4)	0.033 (3)	0.037 (3)	0.093 (4)
C15	0.071 (2)	0.110 (3)	0.202 (6)	0.017 (2)	0.024 (3)	0.076 (4)
C16	0.0577 (16)	0.079 (2)	0.158 (4)	0.0009 (15)	−0.003 (2)	0.056 (2)
C17	0.0680 (18)	0.0517 (15)	0.136 (3)	0.0050 (13)	−0.020 (2)	−0.0096 (17)
C18	0.0648 (17)	0.0672 (18)	0.123 (3)	0.0087 (15)	−0.0039 (18)	−0.0287 (19)
C19	0.0619 (15)	0.106 (2)	0.0696 (17)	−0.0173 (16)	0.0104 (14)	−0.0302 (17)
C20	0.0731 (18)	0.112 (2)	0.0557 (14)	−0.0240 (18)	0.0015 (13)	−0.0057 (16)
C21	0.0556 (14)	0.093 (2)	0.0732 (17)	−0.0043 (14)	−0.0020 (13)	0.0311 (17)
C22	0.0534 (14)	0.0809 (19)	0.0866 (19)	0.0008 (13)	0.0076 (14)	0.0316 (17)
C23	0.0722 (19)	0.096 (2)	0.117 (3)	0.0136 (18)	0.016 (2)	0.045 (2)
C24	0.081 (2)	0.144 (4)	0.150 (4)	0.029 (3)	0.016 (3)	0.085 (4)
C25	0.089 (3)	0.185 (6)	0.115 (3)	0.019 (4)	−0.006 (3)	0.080 (4)
C26	0.0722 (19)	0.140 (3)	0.078 (2)	−0.006 (2)	−0.0080 (17)	0.042 (2)
C27	0.0732 (18)	0.0524 (14)	0.103 (2)	−0.0064 (13)	0.0146 (17)	−0.0025 (15)
C28	0.0644 (15)	0.0657 (16)	0.094 (2)	−0.0155 (13)	0.0059 (15)	−0.0140 (15)
C29	0.0573 (15)	0.088 (2)	0.0756 (18)	−0.0004 (14)	−0.0136 (14)	−0.0204 (15)
C30	0.0702 (18)	0.101 (2)	0.0633 (16)	0.0113 (17)	−0.0067 (14)	−0.0063 (16)
Cl1	0.0875 (6)	0.0753 (5)	0.1399 (9)	0.0048 (4)	−0.0248 (6)	−0.0116 (5)
O21	0.155 (3)	0.099 (2)	0.132 (2)	0.010 (2)	0.018 (2)	−0.0204 (19)
O22	0.135 (3)	0.110 (2)	0.213 (4)	0.006 (2)	−0.068 (3)	−0.051 (3)
O23	0.116 (3)	0.150 (3)	0.180 (4)	−0.009 (2)	0.014 (3)	−0.001 (3)
O24	0.164 (4)	0.122 (3)	0.227 (5)	−0.013 (3)	−0.103 (4)	0.031 (3)
O1W	0.0572 (10)	0.0723 (11)	0.0737 (11)	0.0037 (9)	0.0079 (9)	0.0034 (9)
O2W	0.0788 (13)	0.0803 (13)	0.0782 (13)	−0.0053 (11)	0.0104 (11)	0.0007 (10)
O3W	0.0557 (10)	0.0893 (15)	0.1025 (15)	−0.0050 (10)	0.0010 (10)	−0.0266 (13)
O4W	0.114 (7)	0.121 (7)	0.045 (4)	0.059 (6)	−0.014 (4)	−0.015 (4)

### Geometric parameters (Å, º)

**Table:**

O2—C2	1.220 (4)	C15—H15	0.9300
O6—C6	1.219 (4)	C16—H16	0.9300
N1—C2	1.379 (4)	C17—C18	1.471 (5)
N1—C6	1.379 (4)	C17—H17A	0.9700
N1—H1	0.8600	C17—H17B	0.9700
N3—C4	1.358 (4)	C18—H18A	0.9700
N3—C2	1.388 (4)	C18—H18B	0.9700
N3—C3	1.454 (4)	C19—C20	1.474 (5)
N7—C8	1.315 (5)	C19—H19A	0.9700
N7—C5	1.387 (4)	C19—H19B	0.9700
N7—C7	1.456 (5)	C20—H20A	0.9700
N9—C8	1.334 (5)	C20—H20B	0.9700
N9—C4	1.370 (4)	C21—C26	1.382 (4)
N9—H9	0.8600	C21—C22	1.398 (5)
C3—H3A	0.9600	C22—C23	1.399 (5)
C3—H3B	0.9600	C23—C24	1.415 (7)
C3—H3C	0.9600	C23—H23	0.9300
C4—C5	1.361 (4)	C24—C25	1.360 (8)
C5—C6	1.433 (4)	C24—H24	0.9300
C7—H7A	0.9600	C25—C26	1.355 (8)
C7—H7B	0.9600	C25—H25	0.9300
C7—H7C	0.9600	C26—H26	0.9300
C8—H8	0.9300	C27—C28	1.481 (5)
O11—C11	1.359 (4)	C27—H27A	0.9700
O11—C17	1.428 (4)	C27—H27B	0.9700
O12—C18	1.422 (4)	C28—H28A	0.9700
O12—C19	1.428 (4)	C28—H28B	0.9700
O13—C21	1.351 (4)	C29—C30	1.471 (5)
O13—C20	1.435 (4)	C29—H29A	0.9700
O14—C22	1.370 (4)	C29—H29B	0.9700
O14—C27	1.440 (4)	C30—H30A	0.9700
O15—C28	1.424 (4)	C30—H30B	0.9700
O15—C29	1.430 (4)	Cl1—O23	1.397 (4)
O16—C12	1.377 (4)	Cl1—O24	1.408 (4)
O16—C30	1.418 (4)	Cl1—O22	1.421 (3)
C11—C16	1.375 (4)	Cl1—O21	1.422 (4)
C11—C12	1.388 (5)	O1W—H1O1	0.8201
C12—C13	1.392 (5)	O1W—H2O1	0.8201
C13—C14	1.439 (7)	O2W—H1O2	0.8201
C13—H13	0.9300	O2W—H2O2	0.8199
C14—C15	1.348 (8)	O3W—H1O3	0.8200
C14—H14	0.9300	O3W—H2O3	0.8200
C15—C16	1.330 (8)		
			
C2—N1—C6	128.7 (2)	O12—C18—C17	108.1 (3)
C2—N1—H1	115.7	O12—C18—H18A	110.1
C6—N1—H1	115.7	C17—C18—H18A	110.1
C4—N3—C2	117.7 (3)	O12—C18—H18B	110.1
C4—N3—C3	122.7 (3)	C17—C18—H18B	110.1
C2—N3—C3	119.4 (3)	H18A—C18—H18B	108.4
C8—N7—C5	107.2 (3)	O12—C19—C20	109.7 (2)
C8—N7—C7	125.9 (3)	O12—C19—H19A	109.7
C5—N7—C7	126.8 (3)	C20—C19—H19A	109.7
C8—N9—C4	106.4 (3)	O12—C19—H19B	109.7
C8—N9—H9	126.8	C20—C19—H19B	109.7
C4—N9—H9	126.8	H19A—C19—H19B	108.2
O2—C2—N1	121.5 (3)	O13—C20—C19	106.7 (2)
O2—C2—N3	121.6 (3)	O13—C20—H20A	110.4
N1—C2—N3	116.9 (2)	C19—C20—H20A	110.4
N3—C3—H3A	109.5	O13—C20—H20B	110.4
N3—C3—H3B	109.5	C19—C20—H20B	110.4
H3A—C3—H3B	109.5	H20A—C20—H20B	108.6
N3—C3—H3C	109.5	O13—C21—C26	125.7 (4)
H3A—C3—H3C	109.5	O13—C21—C22	115.2 (3)
H3B—C3—H3C	109.5	C26—C21—C22	119.2 (3)
N3—C4—C5	124.0 (3)	O14—C22—C21	115.4 (3)
N3—C4—N9	127.6 (3)	O14—C22—C23	123.7 (4)
C5—C4—N9	108.4 (3)	C21—C22—C23	120.9 (3)
C4—C5—N7	106.6 (3)	C22—C23—C24	116.6 (5)
C4—C5—C6	121.6 (3)	C22—C23—H23	121.7
N7—C5—C6	131.8 (3)	C24—C23—H23	121.7
O6—C6—N1	122.1 (3)	C25—C24—C23	122.2 (5)
O6—C6—C5	126.9 (3)	C25—C24—H24	118.9
N1—C6—C5	111.0 (3)	C23—C24—H24	118.9
N7—C7—H7A	109.5	C26—C25—C24	120.0 (5)
N7—C7—H7B	109.5	C26—C25—H25	120.0
H7A—C7—H7B	109.5	C24—C25—H25	120.0
N7—C7—H7C	109.5	C25—C26—C21	121.2 (5)
H7A—C7—H7C	109.5	C25—C26—H26	119.4
H7B—C7—H7C	109.5	C21—C26—H26	119.4
N7—C8—N9	111.4 (3)	O14—C27—C28	107.5 (2)
N7—C8—H8	124.3	O14—C27—H27A	110.2
N9—C8—H8	124.3	C28—C27—H27A	110.2
C11—O11—C17	116.9 (3)	O14—C27—H27B	110.2
C18—O12—C19	113.3 (3)	C28—C27—H27B	110.2
C21—O13—C20	116.7 (3)	H27A—C27—H27B	108.5
C22—O14—C27	117.3 (2)	O15—C28—C27	108.9 (2)
C28—O15—C29	113.4 (2)	O15—C28—H28A	109.9
C12—O16—C30	118.1 (3)	C27—C28—H28A	109.9
O11—C11—C16	125.5 (4)	O15—C28—H28B	109.9
O11—C11—C12	115.1 (3)	C27—C28—H28B	109.9
C16—C11—C12	119.4 (4)	H28A—C28—H28B	108.3
O16—C12—C11	115.9 (3)	O15—C29—C30	109.0 (2)
O16—C12—C13	124.0 (4)	O15—C29—H29A	109.9
C11—C12—C13	120.1 (4)	C30—C29—H29A	109.9
C12—C13—C14	116.6 (5)	O15—C29—H29B	109.9
C12—C13—H13	121.7	C30—C29—H29B	109.9
C14—C13—H13	121.7	H29A—C29—H29B	108.3
C15—C14—C13	122.0 (4)	O16—C30—C29	107.8 (2)
C15—C14—H14	119.0	O16—C30—H30A	110.1
C13—C14—H14	119.0	C29—C30—H30A	110.1
C16—C15—C14	119.2 (5)	O16—C30—H30B	110.1
C16—C15—H15	120.4	C29—C30—H30B	110.1
C14—C15—H15	120.4	H30A—C30—H30B	108.5
C15—C16—C11	122.8 (5)	O23—Cl1—O24	108.7 (3)
C15—C16—H16	118.6	O23—Cl1—O22	110.0 (3)
C11—C16—H16	118.6	O24—Cl1—O22	107.1 (3)
O11—C17—C18	107.6 (3)	O23—Cl1—O21	109.5 (3)
O11—C17—H17A	110.2	O24—Cl1—O21	113.2 (3)
C18—C17—H17A	110.2	O22—Cl1—O21	108.4 (2)
O11—C17—H17B	110.2	H1O1—O1W—H2O1	115.1
C18—C17—H17B	110.2	H1O2—O2W—H2O2	104.2
H17A—C17—H17B	108.5	H1O3—O3W—H2O3	110.1

### Hydrogen-bond geometry (Å, º)

**Table:**

*D*—H···*A*	*D*—H	H···*A*	*D*···*A*	*D*—H···*A*
N9—H9···O3*W*	0.86	1.74	2.594 (4)	172
O1*W*—H1*O*1···O12	0.82	2.17	2.941 (3)	158
O2*W*—H1*O*2···O16	0.82	2.41	3.156 (3)	152
O2*W*—H1*O*2···O11	0.82	2.48	3.182 (3)	145
O2*W*—H2*O*2···O14	0.82	2.31	3.124 (3)	175
O3*W*—H1*O*3···O1*W*	0.82	1.99	2.802 (3)	172
O3*W*—H2*O*3···O15	0.82	1.98	2.769 (3)	161
N1—H1···O1*W*^i^	0.86	2.01	2.871 (3)	174
O1*W*—H2*O*1···O2*W*^ii^	0.82	1.93	2.747 (3)	175

Symmetry codes: (i) −*x*+1, *y*+1/2, −*z*+1/2; (ii) *x*−1/2, −*y*+3/2, −*z*.
